# Physiological effects of stress related to helicopter travel in Federal Emergency Management Agency search-and-rescue canines

**DOI:** 10.1017/jns.2017.25

**Published:** 2017-06-07

**Authors:** E. Perry, N. Gulson, T.-W. Liu Cross, K. S. Swanson

**Affiliations:** 1 Department of Animal Science, Food and Nutrition, Southern Illinois University, Carbondale, IL, USA; 2 Division of Nutritional Sciences, University of Illinois, Urbana, IL, USA; 3 Department of Animal Sciences, University of Illinois, Urbana, IL, USA

**Keywords:** Working canines, Stress, Dogs, Travel stress, FEMA, Federal Emergency Management Agency, OTU, operational taxonomic unit

## Abstract

Working canines are deployed by the Federal Emergency Management Agency (FEMA), as part of a National Disaster Response Plan. Stress associated with helicopter flight and the resulting physical effects on the dog are unknown. Our objective was to test the hypotheses that (1) helicopter travel affects the physiology and faecal microbiota of working canines, but that (2) physiological consequences of helicopter travel will not negatively affect their work performance. A total of nine FEMA canines were loaded onto helicopters and flown for 30 min in July 2015. Rectal temperature, behavioural stress indicators and saliva swabs (for cortisol) were collected at baseline, loading, mid-flight and post-flight. After flight, canines completed a standardised search exercise to monitor work performance. Faecal samples were collected for microbial DNA extraction and Illumina sequencing. All canines were on a standardised diet (CANIDAE^®^ Grain Free PURE Land^®^) for 3 weeks prior to the study. Visible indicators of stress were observed at loading and at mid-flight and corresponded with an increase (*P* < 0·05) in salivary cortisol from 5·4 µg/l (baseline) to 6·4 µg/l (loading). Additionally, rectal temperature increased (*P* < 0·05) from 38·61°C (baseline) to 39·33°C (mid-flight) and 39·72°C (post-flight). Helicopter travel did not affect search performance (*P* > 0·05). We found that α- and β-diversity measures of faecal microbiota were not affected (*P* > 0·05). Our data suggest that although helicopter travel may cause physiological changes that have been associated with stress in working dogs, it does not make an impact on their search performance or the stability of faecal microbiota.

Federal Emergency Management Agency (FEMA) canines are often called upon to deploy into scenarios that are unsafe^(^^1^^–^^6^^)^. Helicopters may be required to access areas too remote or with infrastructure too damaged for access by land. It is unclear what effects, if any, this mode of transportation may have upon the canine's performance during disaster response. Although stress from travel has not been well studied, it can cause a wide variety of physiological and psychological health issues in canines, including increased activity of the sympathetic nervous system, increased release of catecholamine and increased blood pressure^(^^7^^)^.

## Stress from travelling

Prior work has reported stress in dogs^(^^8^^)^ for both ground and air transportation. The leading cause of companion animal death during flight has been attributed to over-sedation, followed by respiratory and immunological depression linked to the stress of air travel and finally deaths associated with mishandling of animals in carriers^(^^8^^)^. Although one could certainly argue that search-and-rescue, law enforcement and military canines have been conditioned for work in stressful situations (i.e. perceived by humans as unfavourable)^(^^9^^)^, more investigation is warranted regarding the effects of travel stress on their physiology and performance. Recent work has reported faecal microbiota changes in working canines associated with commercial airline travel with no accompanying change in behaviour or performance^(^^10^^)^. The importance of the work done by these dogs is critical to national security and public safety. An improved understanding of the way stressors affect their health, longevity and performance may facilitate improvements in training and management so as to maximise their welfare.

## Materials and methods

Institutional animal care and use approval was obtained at Southern Illinois University prior to the initiation of this study (no. 14-062). Standards for animal care were adopted from Prescott *et al*.^(^^11^^)^.

### Animals and diets

A total of nine canines were recruited from a FEMA team with a body composition score of 4·5 (sd 0·5) and fed a commercially available complete dry kibble diet for 21 d prior to flight (CANIDAE^®^ Grain Free PURE Land^®^ with bison). Sample size for the study was determined by the number of available dogs meeting the criteria for participation (prior helicopter experience – two flights minimum, no females in heat and completion of certification as type I). Because the dogs were pulled from service for the duration of the study, and due to the very limited number of these types of dogs, nine was the maximum number available for inclusion. Mean body weight for dogs was 28·2 (sd 1·9) kg. Following successful completion of the screening phase, FEMA dogs are trained and tested according to an established federal standard^(^^12^^)^ which includes travel to an unfamiliar location for search work. Canines in our study travelled by vehicle (30 (sd 10) min) to the study site and remained in their temperature-controlled vehicles in kennels (48″ length × 30″ width × 33″ height; 122 cm length × 76 cm width × 84 cm height) except for times of sample collection, flight and search performance. The study took place during July of 2015 in Miami, FL, with all sample collections occurring between 09.00 and 11.00 hours. Sample collection occurred over a 2-d period, with day 1 serving as baseline and day 2 serving as flight day. Canines were housed in their own home environment overnight. Canines had *ad libitum* access to water with the exception of helicopter flight time. Search location was unfamiliar to all canines. Baseline samples were collected at approximately 09.00 hours, including rectal temperature, heart rate, saliva collection (via sterile swab) as well as assessments for stress behaviours as noted by FEMA handlers and two FEMA evaluators^(^^13^^)^. Baseline performance data included total search time, stress behaviours and compliance with handler commands. Temperature, wind speed, and other weather information was monitored and considered seasonally appropriate for the geographic region with minimal change from baseline day to flight day.

Flight occurred in three groups of three canine/handler teams each (based on space and safety considerations). All canine teams were hot-loaded (approach and loading onto the aircraft while the rotors are in motion). This motion creates significant noise and wind, and is commonly utilised in field deployments. Samples collected on flight day were rectal temperature, heart rate and saliva at 15 min pre-flight, loading, 15 min mid-flight and 15 min post-flight. All groups flew for a total of 30 min. Upon landing, canines were immediately unloaded for the standardised search exercise^(^^12^^)^. Dogs must approach and locate the hidden ‘victim’, and perform repetitive barks for 30 s without leaving the victim. Any failure to stay with the victim once barking has begun indicates failure. Inability to locate the victim or complete the 30 s required barking within the allocated 2-min time-frame would indicate failure. Signs of stress, avoidance, successful completion and total search time were recorded. Stool samples were collected from first-morning faecals on day 1 to day 5 for microbial analysis of faecal microbiota.

### Assays

Saliva samples were collected for cortisol measurement with an oral paediatric swab (SalivaBio^®^) and centrifuged for 15 min at 1500 ***g*** (PowerSpin LX™) prior to cortisol analysis (Salivary Cortisol Enzyme Immunoassay Kit; Salimetrics). Saliva cortisol samples were read at 450 nm within 10 min of adding stop solution on a FLUOstar Omega plate reader (BMG Labtech).

Faecal samples were collected in 50 ml sterile vials on ice within 15 min of defecation and frozen overnight prior to overnight shipment (on chemical ice) for laboratory analysis. Upon arriving at the laboratory, samples were stored at −80°C. DNA extraction and purification (Mo Bio PowerSoil kit; MO Bio Laboratories) were followed by quantification (ND-1000 spectrophotometer; Nanodrop™) and verification of DNA quality via gel electrophoresis, all of which were conducted at Southern Illinois University's Chemical Environmental Health and Safety laboratory. 16S rRNA gene amplicons were generated using a Fluidigm Access Array (Fluidigm Corp.) in combination with a Roche High Fidelity Fast Start Kit (Roche). The primers 515F (5′-GTGCCAGCMGCCGCGGTAA-3′) and 806R (5′-GGACTACHVGGGTWTCTAAT-3′) that target a 252-bp fragment of the V4 region were used for amplification (primers synthesised by IDT Corp.). The CS1 forward tag and CS2 reverse tag were added according to the Fluidigm protocol. Quality of amplicons was assessed using a Fragment Analyzer (Advanced Analytics) to confirm amplicon region and size. A DNA pool was generated by combining equimolar amounts of the amplicons from each sample. The pooled samples were then size-selected on a 2 % agarose E-gel (Life Technologies) and extracted using a Qiagen gel purification kit. Cleaned size-selected pooled products were run on an Agilent Bioanalyzer to confirm appropriate profile and average size. Illumina sequencing was performed on a MiSeq using v3 reagents (Illumina Inc.) at the W. M. Keck Center for Biotechnology, University of Illinois. Forward reads were trimmed using the FASTX-Toolkit (version 0.0.13), and QIIME 1.8.0 was used to process the resulting sequence^(^^14^^,^^15^^)^. Briefly, high-quality (quality value >20) sequence data derived from the sequencing process were demultiplexed. Sequences were then clustered into operational taxonomic units (OTUs) using UCLUST through a closed reference OTU picking strategy against the curated GreenGenes 13_8 database^(^^16^^)^, with a 97 % sequence similarity threshold. Singletons (OTUs that were observed fewer than two times) and OTUs that had less than 0·01 % of the total observation were discarded. An even sampling depth (sequences per sample) was used for assessing α- and β-diversity measures. β-Diversity was calculated using weighted and unweighted Unifrac distance measures.

### Statistical procedures

Salivary cortisol and rectal temperature data were analysed using the PROC MIXED procedure of SAS version 9.4 (SAS Institute, Inc.) with results reported as change from baseline with time and dog in the model. Additionally, search performance data were analysed using the PROC TTEST procedure of SAS and were also compared against baseline and are reported as time required to complete the exercise. Principal components analysis of UniFrac distance was calculated for samples based on their 97 % OTU composition to identify any clustering effect for each treatment group. Significance was established at *P* < 0·05.

## Results

Salivary cortisol concentrations were increased mid- and post-flight as a result of helicopter travel (*P* < 0·05) as shown in Table 1. Additionally, rectal temperatures for loading, mid-flight and post-flight values each increased when compared with baseline. Furthermore, mid- and post-flight values exceeded the normal upper values for a canine (39·17°C).

**Table 1.** Physiological effects of stress on nine working canines associated with 30 min of travel by helicopter (Mean values and pooled standard errors)

Parameter	Baseline	Loading	15 min mid-flight	15 min post-flight	sem	*P*
Rectal temperature* (°C)	38·63^a^	39·09^b^	39·34^b^	39·57^b^	0·43	<0·05
Heart rate (beats per min)	96	103	109	97	9·85	>0·05
Salivary cortisol (ng/l)	5·4^a^	5·0^a^	6·5^b^	6·2^b^	1·1	0·02
Total search time† (s)	43	N/A	N/A	41	2·71	0·07

N/A, not applicable.

^a,b^ Mean values within a row with unlike superscript letters were significantly different (*P* < 0·05).

* Normal range 37·78–39·17°C.

† Time required for canine to complete a standardised search task including a 30 s alert.

**Fig. 1.**
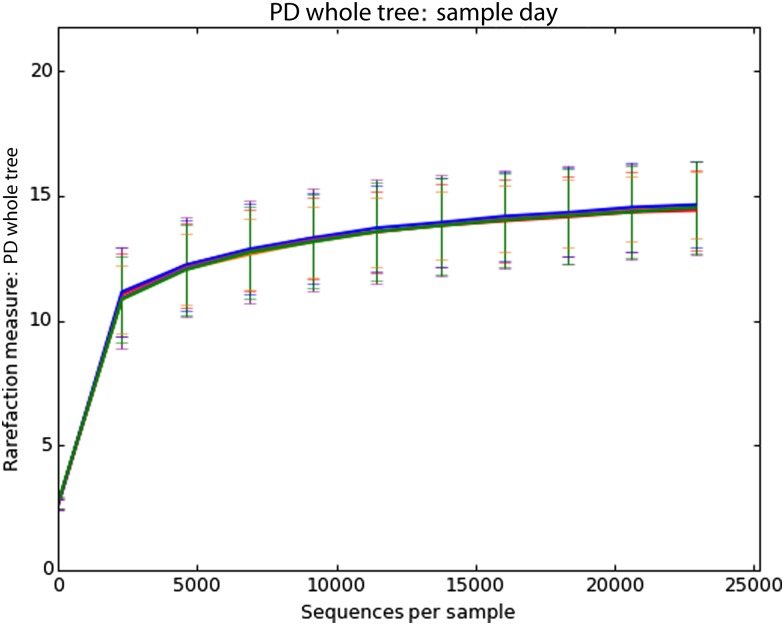
Rarefaction curve depicting no change (*P* > 0·05) in α-diversity of faecal microbiota associated with 30 min of helicopter travel stress in nine working canines. PD, phylogenetic diversity.

Stress behaviours (shaking, refusal to approach, lower posture, tucked tail, panting and tucked ears) were present during loading (five of nine dogs) and mid-flight (two of nine dogs). No change was observed in heart rate (*P* > 0·05) and all values were within the normal range (70–130 bpm). Helicopter travel did not have an impact on performance. No stress behaviours were observed during search and all canines successfully completed the standardised exercise. Helicopter travel did not appear to have an impact on faecal microbiota β-diversity (*P* > 0·05, data not shown) or α-diversity measures (Fig. 1). Additionally, handlers reported no visible change in the faecal scores for the days following helicopter travel.

## Discussion

One common technique for monitoring stress is through the use of the hormone cortisol. Salivary cortisol is reported to peak 15 min post-stimulation^(^^13^^)^. When one considers this delay, it appears that the increase that was observed 15 min into flight may probably be associated with the stress experienced 15 min prior to flight (i.e. during hot-loading). Prior studies have reported that although environmental stress does result in an increase in plasma cortisol, working dogs recover well with no further increases despite follow-up challenges^(^^17^^)^. Other work has shown that dogs responding with elevated salivary cortisol to stimuli demonstrated a return to baseline 45 min after the administration of the stressor^(^^13^^)^. Although the data presented here did not include time points after 15 min post-flight, it seems reasonable (given the lack of behavioural indications) that all dogs recovered within that 45 min period. It is also interesting to point out that the data from our study demonstrate only a moderate increase in cortisol (10–20 %) as compared with other reports as high as 2- to 4-fold^(^^13^^)^. Although the work or training history for the dogs evaluated in the aforementioned study is not identified, it seems likely that the selection criteria coupled with the extensive training associated with successful FEMA dogs would yield a lesser response to environmental challenges (i.e. smaller cortisol changes). Therefore, although the change is smaller than previous studies have reported, it is still significant considering the background of the study participants. Simply put, these dogs have been selected and trained not to react to stressful situations.

Conversely, one must also caution the interpretation of these data. The work performed by these dogs is intense and they are well and continuously trained for their missions. Moderate changes in cortisol, as seen here, although potentially indicative of stress, do not necessarily indicate any long-term adverse physical effects. Another variable that must be considered is the anticipation that the dogs experience. More work needs to be done identifying the biochemical changes associated with excitement as compared with environmental stress.

Few data exist measuring the effect of transport stress on rectal temperature in dogs or the time-frame associated with that elevation. Prior studies examining the effects of exercise on temperature in search-and-rescue dogs have reported increases following 20 min of work^(^^18^^)^. It is unclear what effect, if any, the anticipation of said event would have. It is unknown whether or not there may be effects related to breed or age. Because dogs do not have sweat glands and rely on panting to thermoregulate, any prolonged increase in body temperature that may result in prolonged increased panting is a concern. Panting interferes with olfactory detection^(^^19^^)^ and thus elevations in body temperature associated with travel stress are a significant concern for working dogs who rely on their olfactory abilities to perform their jobs. Panting and heart rate elevations shown as a result of stress in prior studies may also be explained due to lack of physical conditioning. Improved cardiovascular condition may explain why the dogs in the present study showed no difference in heart rate as compared with the laboratory dogs used in a prior study where heart rate was increased^(^^13^^)^.

Behavioural indicators of stress have been well documented in companion dogs^(^^13^^,^^20^^,^^21^^)^. Literature regarding signs of stress in working dogs is available^(^^22^^–^^24^^)^; however, there are few data assessing their performance following environmental stress. One study examining the effects of commercial air travel on working canines reported no behavioural indicators of stress or impact on performance associated with flight^(^^10^^)^.

The tendency toward improvement in total search time is intriguing. Research conducted in human subjects has reported that highly trained and skilled athletes were markedly less reactive to stressors than their untrained counterparts^(^^25^^)^. Anecdotal reports from the handlers participating in the study indicated that the dogs seemed to ‘calm down’ when they were given the search command. It is possible that the familiarity of the command and the training associated with that work allowed the dogs to focus on the task and disassociate themselves from the environmental stress previously experienced. It is also possible that this was simply improvement relative to the previous day. Future studies should include measurements taken over several days in order to minimise effect of day.

There are few data available regarding the effects of chronic stress on the working dog microflora and it appears that the acute nature of the stress applied in this test (i.e. hot-loading followed by 30 min of flight) was insufficient to result in a change in the faecal microbiota. In a previous study, working canines transported via commercial airline had differences in faecal microbiota despite the fact that no outward signs of stress were observed^(^^10^^)^. However, the other physiological data suggested that the dogs were stressed. It may simply be that the nature of commercial airline travel stress (occurring over many hours with unfamiliar environment) is sufficient to cause a change in the gut microbiota but the short-lived stress of helicopter travel is not.

There were some limitations to our study. Although all dogs had indicated prior helicopter experience (minimum two flights) there may be differences associated with frequency. Length of time in the system and training exposure may yield differences in response. Although all dogs had completed standardised training and were certified as ready for deployment, individual training associated with age may have an impact on results. Unfortunately, the limited number of dogs available for these types of studies can cause some difficulty. Differences associated with breed and deployment history may also contribute to factors which can make an impact on results.

Although the lack of a control group may be viewed as a limitation, it should also be considered that comparison with one's own baseline may provide the best capture of any changes that occurred. Recent work indicates effects on cortisol related to sex, neuter status, age, home environment, time in environment prior to sample collection, owner's presence while testing, media used for collection, differences between dogs as well as individual variation^(^^26^^)^. Age, sex, neuter status and individual variation were not factors that we could control with the number of dogs available. Since the baseline values for each dog served as their own control, the comparison back to self helped to eliminate variation arising between dogs.

Helicopter travel is often utilised on mission. It is critical to understand how this affects both the health and performance of dogs. Because the success of search-and-rescue canines is often measured in human lives, more research in this area is justified.

## References

[1] Otto CM, Franz BK, Lewis R, (2002) Field treatment of search dogs: lessons learned from the World Trade Center disaster. Vet Emerg Crit Care Soc 12, 33–42.

[2] Jones K, Dashfield K, Downend A, (2004) Search-and-rescue dogs: an overview for veterinarians. J Am Vet Med Assoc 225, 854–860. 15485043 10.2460/javma.2004.225.854

[3] Slensky K, Drobatz K, Downend A, (2004) Deployment morbidity among search-and-rescue dogs used after the September 11, 2001, terrorist attacks. J Am Vet Med Assoc 225, 868–873. 15485045 10.2460/javma.2004.225.868

[4] Fitzgerald S, Rumbeiha W, Braselton W, (2008) Pathology and toxicology findings for search-and-rescue dogs deployed to the September 11, 2001, terrorist attack sites: initial five-year surveillance. J Vet Diagn Invest 20, 477–484. 18599853 10.1177/104063870802000410

[5] Gordon L (2012) Injuries and illnesses among urban search-and-rescue dogs deployed to Haiti following the January 12, 2010 earthquake. J Am Vet Med Assoc 240, 396–403. 22309011 10.2460/javma.240.4.396

[6] Gordon L (2015) Injuries and illnesses among Federal Emergency Management Agency-certified search-and-recovery and search-and-rescue dogs deployed to Oso, Washington, following the March 22, 2014 State Route 530 landslide. J Am Vet Med Assoc 247, 901–908. 26421402 10.2460/javma.247.8.901

[7] Scholz M & von Reinhardt C (2007) Stress in Dogs. Wenatchee, WA: Dogwise Publishing.

[8] Bergeron R, Scott SL, Emond JP, (2002) Physiology and behavior of dogs during air transport. Can J Vet Res 66, 211–216. 12146895 PMC227007

[9] Helton WS (2009) Canine Ergonomics: The Science of Working Dogs. Boca Raton, FL: CRC Press.

[10] Venable E, Bland S, Holscher H, (2016) Effects of air travel stress on the canine microbiome: a pilot study. Int J Vet Health Sci Res 4, 132–139.

[11] Prescott M, Morton D, Anderson D, (2004) Refining dog husbandry and care. Eighth report of the BAAWF/FRAME/RSPCA/UFAW joint working group on refinement. Lab Anim 1, 1–94. 10.1258/002367704323145742 15202954

[12] Federal Emergency Management Agency (FEMA) (2008) Canine Search Specialist Certification Process. National Urban Search and Rescue Response System, Department of Homeland Security, Federal Emergency Management Agency, 38. www.fema.gov/emergency/usr/ (accessed May 2015).

[13] Beerda B, Schilder MB, van Hooff J, (1998) Behavioural, saliva cortisol and heart rate responses to different types of stimuli in dogs. Appl Anim Behav Sci 58, 365–381.

[14] Caporaso JG, Kuczynski J, Stombaugh J, (2010) QIIME allows analysis of high-throughput community sequencing data. Nat Methods 7, 335–336. 20383131 10.1038/nmeth.f.303 PMC3156573

[15] Holscher HD, Bauer LL, Gourineni V, (2015) Agave inulin supplementation affects the fecal microbiota of healthy adults participating in a randomized, double-blind, placebo-controlled, crossover trial. J Nutr 145, 2025–2032. 26203099 10.3945/jn.115.217331

[16] DeSantis TZ, Hugenholtz P, Larsen N, (2006) Greengenes, a chimera-checked 16S rRNA gene database and workbench compatible with ARB. Appl Environ Microbiol 72, 5069–5072. 16820507 10.1128/AEM.03006-05 PMC1489311

[17] Haverbeke A, Diederich C, Depiereux E, (2008) Cortisol and behavioral responses of working dogs to environmental challenges. Phys Behav 93, 59–67. 10.1016/j.physbeh.2007.07.014 17868751

[18] Rovira S, Munoz A & Benito M (2008) Effect of exercise on physiological, blood and endocrine parameters in search and rescue-trained dogs. Veter Med 53, 333–346.

[19] Gazit I & Terkel J (2003) Explosives detection by sniffer dogs following strenuous physical activity. Appl Anim Behav Sci 2, 149–161.

[20] Beerda B, Schilder M, van Hooff J, (1997) Manifestations of chronic and acute stress in dogs. Appl Anim Behav Sci 52, 307–319.

[21] Beerda B, Schilder M, van Hooff J, (2000) Behavioral and hormonal indicators of enduring environmental stress in dogs. Anim Welf 9, 49–62.

[22] Burghardt WF (2003) Behavioral considerations in the management of working dogs. Vet Clin Small Anim 33, 417–446. 10.1016/s0195-5616(02)00133-x 12701519

[23] Foyer P, Svedberg A, Nilsson E, (2016) Behavior and cortisol responses of dogs evaluated in a standardized temperament test for military working dogs. J Vet Behav 11, 7–12.

[24] Wilsson E & Sundgren P-E (1997) The use of a behavior test for the selection of dogs for service and breeding, I: method of testing and evaluating test results in the adult dog, demands on different kinds of service dogs, sex and breed differences. Appl Anim Behav Sci 53, 279–295.

[25] Rimmele U, Zellweger B, Marti B, (2007) Trained men show lower cortisol, heart rate and physiological responses to psychosocial stress compared with untrained men. Psychoneuroendocrinology 32, 627–635. 17560731 10.1016/j.psyneuen.2007.04.005

[26] Cobb ML, Iskandarani K, Chinchilli V & Dreschel NA (2016) A systematic review and meta-analysis of salivary cortisol measurement in domestic canines. Domest Anim Endocrinol 57, 31–42. 27315597 10.1016/j.domaniend.2016.04.003

